# Triazolo Based-Thiadiazole Derivatives. Synthesis, Biological Evaluation and Molecular Docking Studies

**DOI:** 10.3390/antibiotics10070804

**Published:** 2021-07-02

**Authors:** Charalampos Kamoutsis, Maria Fesatidou, Anthi Petrou, Athina Geronikaki, Vladimir Poroikov, Marija Ivanov, Marina Soković, Ana Ćirić, Alejandro Carazo, Přemysl Mladěnka

**Affiliations:** 1 School of Pharmacy, University of Patras, 26504 Patras, Greece; kamoutsi@upatras.gr; 2 School of Pharmacy, Aristotle University of Thessaloniki, 54124 Thessaloniki, Greece; marifesa@pharm.auth.gr (M.F.); aipetrou@pharm.auth.gr (A.P.); 3 Institute of Biomedical Chemistry, Laboratory of Structure-Function Drug Design, Pogodinskaya str. 10, Bldg. 8, 119121 Moscow, Russia; vladimir.poroikov@ibmc.msk.ru; 4 Institute for Biological Research “Siniša Stanković”—National Institute of Republic of Serbia, University of Belgrade, Blvd. Despot Stefan 142, 11000 Belgrade, Serbia; marija.smiljkovic@ibiss.bg.ac.rs (M.I.); mris@ibiss.bg.ac.rs (M.S.); rancic@ibiss.bg.ac.rs (A.Ć.); 5 Department of Pharmacology and Toxicology, Faculty of Pharmacy, Charles University, Akademika Heyrovského 1203, 500 05 Hradec Králové, Czech Republic; carazofa@faf.cuni.cz (A.C.); mladenkap@faf.cuni.cz (P.M.)

**Keywords:** thiadiazole derivatives, triazole, antimicrobial, antifungal, biofilm, docking, toxicity

## Abstract

The goal of this research is to investigate the antimicrobial activity of nineteen previously synthesized 3,6-disubstituted-1,2,4-triazolo[3,4-b]-1,3,4-thiadiazole derivatives. The compounds were tested against a panel of three Gram-positive and three Gram-negative bacteria, three resistant strains, and six fungi. Minimal inhibitory, bactericidal, and fungicidal concentrations were determined by a microdilution method. All of the compounds showed antibacterial activity that was more potent than both reference drugs, ampicillin and streptomycin, against all bacteria tested. Similarly, they were also more active against resistant bacterial strains. The antifungal activity of the compounds was up to 80-fold higher than ketoconazole and from 3 to 40 times higher than bifonazole, both of which were used as reference drugs. The most active compounds (**2**, **3**, **6**, **7**, and **19**) were tested for their inhibition of *P. aeruginosa* biofilm formation. Among them, compound **3** showed significantly higher antibiofilm activity and appeared to be equipotent with ampicillin. The prediction of the probable mechanism by docking on antibacterial targets revealed that *E. coli* MurB is the most suitable enzyme, while docking studies on antifungal targets indicated a probable involvement of CYP51 in the mechanism of antifungal activity. Finally, the toxicity testing in human cells confirmed their low toxicity both in cancerous cell line MCF7 and non-cancerous cell line HK-2.

## 1. Introduction

Despite an indisputable contribution of the existing antimicrobial agents to life expectancy, bacterial infections continue to cause serious diseases which lead to mortality in all parts of the world. The main reason for this is antimicrobial resistance, which is the result of the appearance and broad extension of microbes including both Gram-positive and Gram-negative bacteria [1,2,3,4,5]. The main targets of antimicrobial drugs are the biosynthesis of proteins, RNA, DNA, cell walls, and folic acid. Indeed, numerous inhibitors against them have been successfully discovered [6,7]. Nevertheless, the rate of novel antibiotic discoveries is markedly diminished compared to the period referred to as the “golden era” of antibiotic drug discovery [8].

On the other hand, another fundamental problem is invasive and systemic fungal infections, which are also complicated by the development of resistant strains in health care units nowadays. This has led to a rise in death, mostly due to the Candida and Aspergillus species [9,10]. Immune-deficient patients and those using often antimycotic drugs are more susceptible to infections caused by these two species. 

In the past decades, the problem of multidrug resistant microorganisms has reached a dangerous level around the world, causing serious life-threatening infections. Thus, the problem of multidrug-resistant bacteria and the fight against them is still, and likely even more so than in the past, an attractive target for the scientific community. The development of new molecules with different and particularly dissimilar mechanisms of action, circumventing cross-resistance relative to current accessible therapeutics, is highly desired.

Heterocycles with nitrogen and sulfur (in particular five membered with two or three heteroatoms) are structural units in many pharmaceutical preparations, encompassing antifungal drugs used for invasive infections such as fluconazole, intraconazole, viroconazole, and posaronazole, and anti-glaucoma and antileptic drugs blocking carbonic anhydrase acetazolamide and methazolamide. Thus, 1,2,4-triazoles and their heterocyclic derivatives represent attractive agents with numerous biological properties such as antitubercular [11,12,13], analgesic [14,15], anti-inflammatory [14,16,17], anticancer [18], anticonvulsant [19,20], antiviral [21], antibacterial [22,23,24,25], and antifungal properties [26,27,28,29].

A close structural alternative is the 1,3,4-thiadiazole ring. Being a part of the latest generation of cephalosporins, it encourages scientists to utilize this scaffold in the preparation of new antimicrobial agents. This has stimulated investigation for using this ring in the preparation of novel antimicrobial compounds. Drugs containing this ring are known to have multiple biological activities such as antimicrobial [30,31,32], antifungal [33,34,35], and a range of other pharmacological properties [36,37,38,39,40].

Similarly, sulfonamides have attracted the interest of researches due to their wide spectrum of biological activity, including known antibiotics [41] but also antitumor drugs [42,43], carbonic anhydrase inhibitors [44,45,46], anti-inflammatory drugs [46,47], antiretroviral activity [48,49], and, of course, antimicrobial properties [50,51,52,53,54,55] among others [56,57,58,59].

It should be mentioned that sulfonamides were the first antibiotics ever clinically used and, since that time, they are still frequently employed. They are known to be active against both Gram-positive and Gram-negative bacteria. Due to low manufacturing cost, combined with potent activity against bacterial illnesses, sulfonamides and their various derivatives are among the most widely used antimicrobial agents [60].

We have synthesized previously nineteen new compounds that combine in the structure triazolo based-thiadiazole and sulfonamide moieties. Thus, they may exhibit potent antimicrobial action. The combination of two or more bioactive pharmacophores in one frame is essential for the new drug discovery [61].

The purpose of this study is the experimental testing of the antibacterial and antifungal actions of the synthesized compounds, which was conducted in order to identify the most promising antimicrobial agents and determine which activity should be further evaluated in greater detail.

## 2. Results and Discussion

### 2.1. Chemistry

All compounds were previously synthesized by us, and their synthetic scheme and characterization was presented in our previous paper [62]. Their structures are presented in Table 1.

### 2.2. Prediction of Toxicity

Considering the importance of predicting toxicity in drug design, two computer programs, ToxPredict (OPENTOX) and PROTOX, were employed in the current work [63,64,65]. These programs predict probability of carcinogenicity and mutagenicity in various organisms using in silico models and the semi lethal dose (LD_50_) in rodents. The accuracy of prediction increases as the confidence values rise. In particular, reliable estimates should be higher than 0.025. All compounds showed a confidence from 0.026 to 0.041 and an LD_50_ of 800 mg/kg or higher belonging to group four, according to the Globally Harmonized System of Classification and Labeling of Chemicals (GHS) [66], and were considered safe for biological experiments. The results of the prediction are presented in Tables S1 and S2 (Supplementary Files).

### 2.3. Biological Evaluation

#### 2.3.1. Antibacterial Activity

Compounds **1**–**19** were evaluated for their antibacterial activity by a microdilution method. The minimum inhibitory concentration of compounds was between 5 and 150 μg/mL and MBC from 10 to 200 μg/mL (Table 2).

The antibacterial potency can be presented as: **19** > **3** > **6** > **2** > **7** > **9** = **10** = **11** = **12** > **15** > **18** > **5** = **17** > **8** > **16** > **4** > **13** > **14** > **1**. The most potent antibacterial activity was achieved for the derivative **19** with MIC in range of 5–20 μg/mL and MBC at 10–40 μg/mL, whereas compound **1** was the least active. It was observed that the sensitivity of most bacteria to the studied derivatives was almost similar. Thus, the effectiveness of compounds tested against *E. coli* can be presented as follows: **1** = **2** = **3** = **5** = **9** = **11**= **12** = **16** = **17** = **18** > **6** = **10** = **14** = **15** > **4** = **7** > **8** > **13** > **19**, while for the most resistant *P. aeruginosa* it was: **1** = **3** = **5** = **7** = **13** > **2** = **6** = **9** = **19** > **4** > **10** = **11** = **15**= **18** > **8**= **14** = **17** > **12** = **16**.

Compounds **17** and **19** exhibited the best activity against *B. cereus* with MIC at 8 mg/mL, 5 μg/mL, and MBC at 20 μg/mL and 10 mg/mL, respectively. Good activity against this bacterial strain was also shown by compounds **3**, **4**, **5**, **7**, **10**, **14**, **16**, and **18** with MIC values of 15 μg/mL and MBC at 20 μg/mL, while some of them (**3**, **5** and **7**) displayed the similar good activity against *P. aeruginosa*. Compounds **6**, **19** (MIC 5 μg/mL, MBC 10 μg/mL) and **10** with MIC at 0.008 mg/mL exhibited very good activity against *S. Typhimurium*, while compounds **1** and **2** also showed good activity against this strain. Compound **3** exhibited the best activity against *L. monocytogenes* (MIC 5 μg/mL), whereas *S. aureus* was mostly susceptible to derivatives **2**, **4**, **6**, **10**, and **19** (MIC 10 μg/mL).

It is interesting to mention that all derivatives demonstrated higher activity than both reference drugs, ampicillin and streptomycin, against the examined bacterial strains (Table 2).

Analysis of the structure-activity relationships demonstrated that the presence of benzene (**19**) as substituent at the position 6 of 1,2,4-triazolo-[3,4-b]-1,3,4-thiadiazole group is favorable for antibacterial activity. Replacement of benzene by cinnamic acid (**3**) mildly reduced the activity, while the introduction of phenoxymethyl as a substituent at position 6 (**6**) decreased the activity more significantly. The presence of 3,4-dimethoxy-benzyl (**2**) appeared to be less important than the two previous ones, being still (like compounds **3** and **6**) among the most active compounds, while the 4-methoxy-benzyl group (**1**) had a negative influence on the activity.

The activity of all compounds against resistant strains was also investigated (Table 3). The antibacterial effects against three selected resistant strains of bacteria (MRSA, *P. aeruginosa* and *E. coli*) were completely different than that against non-resistant strains and followed the order: **11** > **17** > **10** > **1** > **16** > **19** > **9** > **7** > **4** > **3** > **6** > ¬ **12** = **13** > **2** > **5** > **18** > **8** > **15** > **14**. Thus, the most potent compound against resistant strains appeared to be **11** with MIC (8–10 μg/mL) and MBC values of 10 to 20 μg/mL, while towards the non-resistant strains it was in the middle of the activity order. Compound **1** showed the lowest activity against non-resistant strains, while against resistant ones it was one of the most active. Like in case of non-resistant bacteria the most sensitive among resistant strains appeared to be *E. coli* and the most resistant was *P. aeruginosa.*

Ampicillin exhibited only an inhibitory potency at 200 μg/mL, Streptomycin possessed MIC at 50–100 μg/mL and MBC at 100–200 μg/mL, with no bactericidal effect observed against MRSA. Hence, the tested compounds provided superior activity over ampicillin and streptomycin.

From the study of the structure-activity relationships, it is obvious that the presence of 2-chloro-4-nitrobenzene (**11**) at the position 6 of 1,2,4-triazolo-[3,4-b]-1,3,4-thiadiazole group was favorable for antibacterial activity against resistant strains, while the introduction of 3-amino-benzene (**17**), 3-methyl-4-nitrobenzene (**10**), and phenoxymethyl **(1**) as substituents led to compounds with decreased activity. On the other hand, the presence of the 4-pyridinyl (**14**) substituent was detrimental on activity against resistant bacteria, resulting in a less active compound.

The comparison of activity toward non-resistant and resistant strains demonstrated that there are large differences. Thus, the compound **19** was the most potent against non-resistant strains, while it was less active against resistant bacteria being in position 6 of the activity order. On the other hand, compound **11** was the most active against resistant strains and was not very active against non-resistant ones.

The comparison of activity of aminophenyl derivatives (**7, 17** and **18**) revealed that the most favorable activity for non-resistant strains was shown by 2-aminophenyl (**7**), followed by 4-aminophenyl (**18**), with the 3-aminophenyl substitution (**17**) to be the less potent one, while for resistant strains it is **17** > **7** > **18**. In the case of methoxybenzyl derivatives (**1**, **2**, **8**, **9**), the order of activity can be presented as **2** > **9** > **8** > **1**. Thus, the most potent among them was found to be 2,3-dimethoxybenzene (**2**) and lower potential was observed for 4-methoxybenzyl substitution (**1**), while in instance of resistant strains compound **1** was the most active (**1** > **9** > **2** > **8**). Among pyridine substituted derivatives (**14, 15, 16**), the most active appeared to be the 2-pyridine derivative (**15**), and compound **14** the less active, while against the resistant strains the order was **16** > **15** > **14**.

Five compounds at the MIC and its half (**2, 3, 6, 7, 19**) were tested for their ability to inhibit *P. aeruginosa* biofilm formation. The biofilm formation is a means of self-protection of bacteria, which often leads to decreasing the therapeutic effects and increasing the drug resistance. More importantly, biofilm accounts for more than 70% of human bacterial infection, which causes a series of obstacles to antibacterial treatment [67].

MIC of compound **3** showed significantly higher antibiofilm activity compared to other compounds (Table 4) and was more potent than ampicillin and streptomycin. Compound **3** applied even in concentration twice lower than its MIC still exhibited promising antibiofilm activity and reduced *P. aeruginosa* biofilm for 62.82%. The remaining compounds (**2, 6, 7** and **19**), applied in their MICs and 0.5 MICs showed similar reduction abilities (44.13–50.85% and 31.9–41.5%, respectively).

#### 2.3.2. Antifungal Activity

Evaluation of antifungal activity of triazolo-thiadiazole derivatives revealed that all compounds displayed very good antifungal activities (MIC at 2–40 μg/mL and MFC at 5–67 μg/mL, Table 5) with the following order: **4** > **6** > **2** > **16** > **1** > **7** > **8** > **18** > **14** > **15** > **17** > **3** > **19** > **9** = **10** > **11**> **5** > **13** > **12**. It is obvious that compound **4** demonstrated the highest potency among all derivatives with MIC values in range of 2–10 μg/mL and MFC at 5–20 μg/mL. On the other hand, compound **12** showed the lowest activity. Interestingly, this compound was one of the less active against bacteria too.

Similar to the bacteria, most of the fungi showed analogous sensitivity towards the triazolo-thiadiazoles tested. Therefore, the order of activity, against the most sensitive *T.viride*, can be presented as: **1** = **2** = **3** = **6** = **11** = **13** > **16** > **4** = **7** = **8** = **9** = **10** = **12** = **14** = **15** = **17** > **5** = **18** = **19**, while against two of the most resistant fungi *P. funiculosum* and *P. verrucosum var. cyclopium* it was: **11** > **7** > **2** = **4** = **6** = **8** = **10** = **16** > **1** = **3** = **18** = **19** > **5** = **14** = **15**= **17**> **12** > **8** = **13** and **11** > **1** = **2** = **4** = **6** > **8** > **3** = **5** = **7**= **10** = **12** = **16** = **19** > **9** = **13** = **14** = **15** = **17** = **18**, respectively.

Compounds, **1, 3, 6, 11** and **13** demonstrated excellent activity against *T. viride* (MIC/MFC at 2/5 μg/mL). Additionally, derivative **11** had the same potency also against *P. funiculosum* and *P. verrucosum var. cyclopium,* while compounds **1** and **4** against *A. versicolor*. Compounds **6** and **9** exhibited good activity (MIC 5 μg/mL, MFC 10 μg/mL) against filamentous *A. versicolor* and *A. fumigatus* fungi. These strains cause aspergillosis and, together with candidiasis, are mostly responsible for morbidity and mortality of *immunocompromised patients* [68].

Ketoconazole demonstrated antifungal potency at MIC 200–1000 μg/mL and MFC at 500–1500 μg/mL, being 80-fold less active than the triazolo-thiadiazole derivatives, whereas bifonazole showed MIC at 100–200 μg/mL and MFC at 200–50 μg/mL, 3–40 times less than that of studied compounds.

Analysis of the structure-activity relationships revealed that for antifungal activity, the presence of 2-(2-methoxy-phenyl)-ethyl group in position 6 of triazole-thiadiazole plays a positive role since compound (**4**) exhibited the best activity. Replacement of this substituent by phenoxymethyl (**6**), 3,4-dimethoxy-benzyl (**2**) and 3-bromo-pyridinyl (**16**) groups decreased activity but these compounds still remained among the most active. The introduction of a benzyl group at the position 6 was not favorable in relation to antifungal activity. The obtained results showed that compounds **2** and **6** exhibited dual action, both antibacterial and antifungal.

In conclusion, all compounds demonstrated good antibacterial against non- and resistant strains, as well as antifungal potency higher than the reference drugs ampicillin, streptomycin, ketoconazole, and bifonazole.

### 2.4. Docking Studies

#### 2.4.1. Docking to Antibacterial Targets

According to the estimated energies of binding to *E. coli* DNA gyrase, thymidylate kinase, *E. coli* primase, and *E. coli* MurA it is obvious that they are higher than that to *E. coli* MurB. Thus, it seems that *E. coli* MurB is probably involved in the mechanism of antibacterial activity (Table 6).

**Figure 1:**
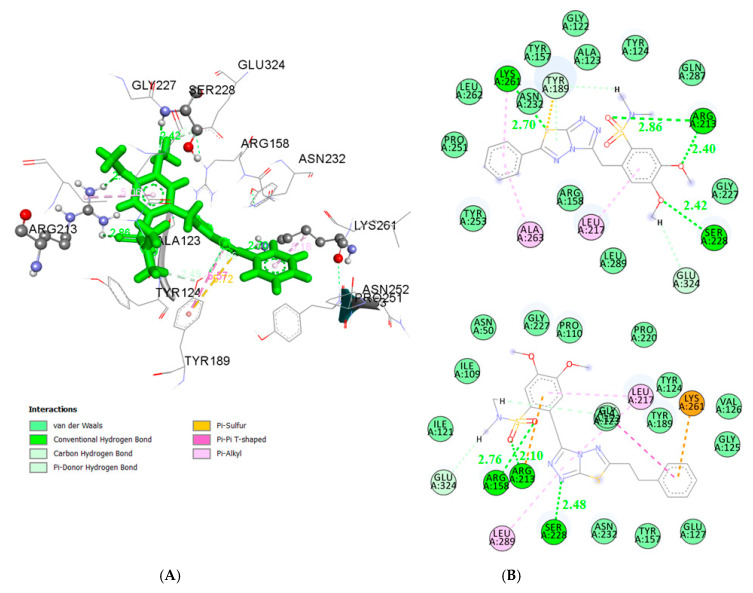
(**A**) Docked conformation of the most active compound **19** in *E.coli* MurB; (**B**) 2D diagrams of the most active compounds **19** (up) and **3** (down) in *E.coli* MurB.

According to docking pose of the most active compound **19** in the *E. coli* MurB enzyme, four favorable hydrogen bond interactions were observed. They are between the oxygen atom of –one OCH_3_ group of the compound and the hydrogen of the side chain of Ser228, and the oxygen atom of the other -OCH_3_ group and the side chain of Arg213 (distance 2.24 Å and 2.40 Å, respectively), as well as between the oxygen atom of the –SO_2_ group of the compound and Arg231 and the S atom of the compound and Lys261 (distance 2.89 Å and 2.70 Å, respectively). Hydrophobic interaction of the fused rings and the residues Ala123, Tyr189, Asn232, Tyr157, and Arg158 as well as of the substituted benzene and the residues Tyr124, Gln287, Gly227, Glu324, Leu289, and Leu217 were observed (Figure 1). On the other hand, the second benzene ring is placed into a cavity consisting of the residues Leu262, Pro251, Tyr253, and Ala263 displaying hydrophobic interaction which contributed to the stabilization of the compound-enzyme complex, justifying the high activity of derivative **19**. It is important to highlight the role of hydrogen bond with the residue Ser228, which is involved in the proton transfer at the second stage of peptidoglycan synthesis [69]. The formation of the above-mentioned hydrogen bond by compound 3 also explains its high inhibitory action (Figure 1). It should be mentioned that these compounds, according to the docking studies, inhibit MurB enzyme almost in a similar way forming a hydrogen bond with the residue Ser228 as 3,5-dioxopyrazolidines reported by Yang et al. [70], as well as the thiazolidinones derivatives of our previous work [71].

**Figure 2:**
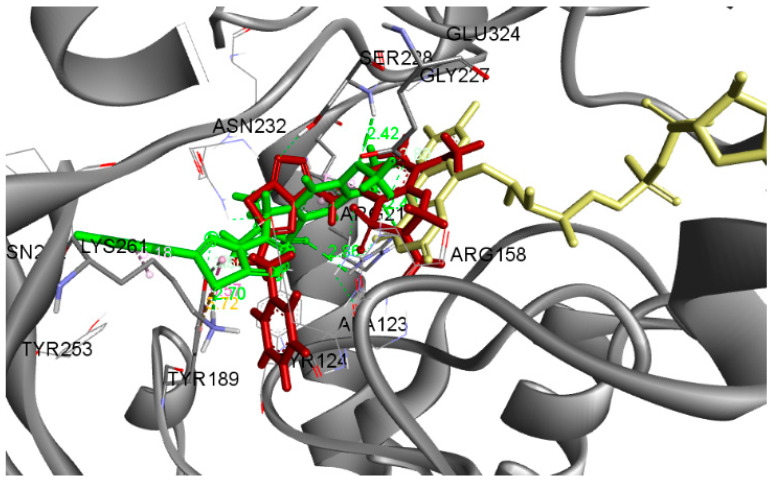
Docked conformation of compounds **19** (green), **3** (red), and FAD (yellow) in *E.coli* MurB.

The docking results indicated that compounds **3** and **19** bind MurB in a similar way, fitting into the binding center of the enzyme due to the formation of H-bond with Ser228 (Figure 2).

#### 2.4.2. Docking to Antifungal Targets

Docking of the thiazolo-triazole derivatives as well as ketoconazole was performed on the DNA topoisomerase and 14α-demethylase of *C. albicans (*Table 7). The last one is the main target of known antifungal drugs.

**Figure 3:**
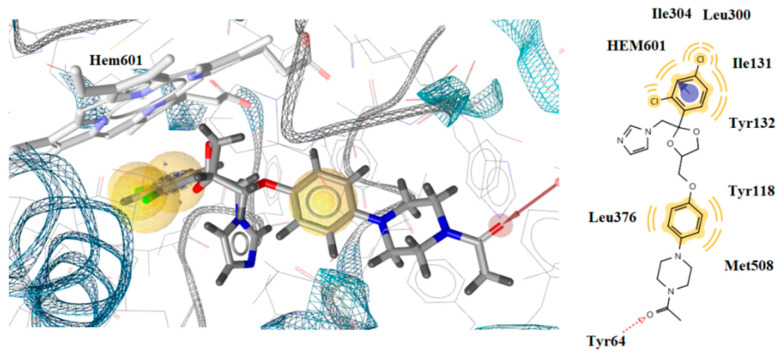
Docked conformation of ketoconazole in lanosterol 14α-demethylase of *C*. *albicans* (CYP51_ca_).

**Figure 4:**
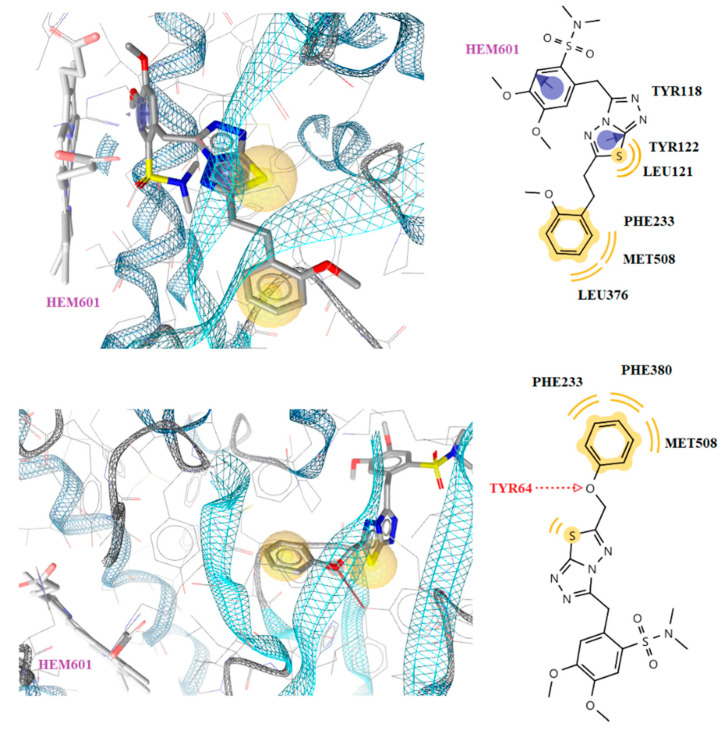
Docked conformation of compound **4** (up) and **6** (down) in lanosterol 14α-demethylase of *C*. *albicans* (CYP51_ca_).

It was observed that the most active compound **4** was placed inside the enzyme alongside the heme group, forming aromatic interaction of benzene ring with CYP51Ca as well as hydrophobic interactions between Phe233, Leu376, and Met508 and the benzene rings of the compound. Despite the formation of aromatic interaction of ketoconazole benzene ring with heme, it lacks the hydrophobic interaction detected in compound **4** (Figure 3 and Figure 4). On the other hand, although no interaction of compound **6** with the heme group was observed, a hydrogen bond formation between the oxygen atom of the side chain of the compound and the hydrogen atom of the side chain of Tyr64 was detected, similar to ketoconazole (Figure 3 and Figure 4).

### 2.5. Search for Structural Analogs

We performed the search for structural analogs of the 19 compounds in the CDDI database [72]. As a result, we found that the structural formulae of five compounds investigated in our study match those described earlier [73]. The correspondence between the molecules is as follows: **1** (TS-50), **7** (TS-57), **15** (TS-66), **18** (TS-71), and **19** (TS-167), where the identifiers given in parenthesis are from the paper [71]. However, all five compounds were previously studied in cell cultures as potential oncolytic drugs. No antibacterial and antifungal activity was investigated for those compounds. 

Also, for all five compounds, acute toxicity data were determined as LD50 > 500 mg/kg (mouse C57BL/6, intraperitoneally) [73], which are in agreement with our predictions given in Table S2.

It is necessary to highlight that no other structural analogs with antimicrobial activity were found in the CDDI, which provides evidences for the novelty of the tested compounds in this field [74,75].

### 2.6. In-Silico Predictive Studies

Drug likeness is examined as an important part that provides the base for the molecules to be a powerful oral drug candidate. Various rules viz. Lipinski, Ghose, Veber, Egan, and Muegge were considered to calculate drug-likeness of the candidate compounds.

**Figure 5:**
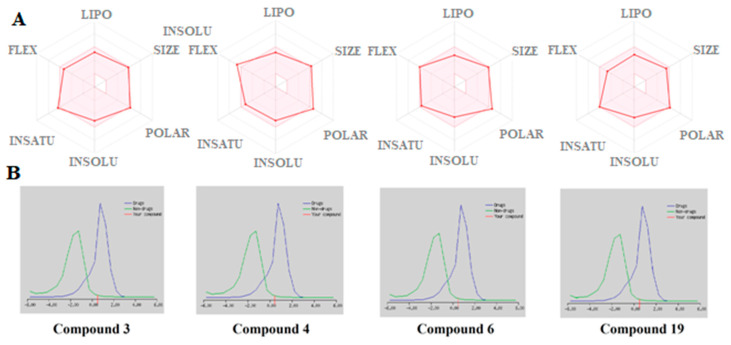
(**A**) Bioavailability Radar of the tested compounds. The pink area represents the optimal range for each property for oral bioavailability, (Lipophilicity (LIPO): XLOGP3 between −0.7 and +5.0, Molecular weight (SIZE): MW between 150 and 500 g/mol, Polarity (POLAR) TPSA between 20 and 130 Å^2^, Solubility (INSOLU): log S not higher than 6, Saturation (INSATU): fraction of carbons in the sp3 hybridization not less than 0.25, and Flexibility (FLEX): no more than 9 rotatable bonds. (**B**) Drug-likeness model score.

The results (Table 8) revealed that most of the compounds violated any rule and their bioavailability score was around 0.55, except for compounds **2**, **9**, **10**, and **11** which had two violations and a bioavailability score 0.17. All compounds exhibited moderate to good drug-likeness scores in the range from −0.34 to 0.94, with compounds **13** and **14** exhibiting the best drug-likeness score with values 0.94 and 0.89, respectively. In the case of the most actives compounds in accordance to biological experiments compounds **3**, **4**, **6**, and **19** appeared to have a good in silico prediction with a good drug-likeness score with a value ranging from 0.45 to 0.53. The bioavailability radar of these compounds is displayed in Figure 5, along with their Drug-likeness model score.

### 2.7. Cytotoxicity Assays

**Figure 6:**
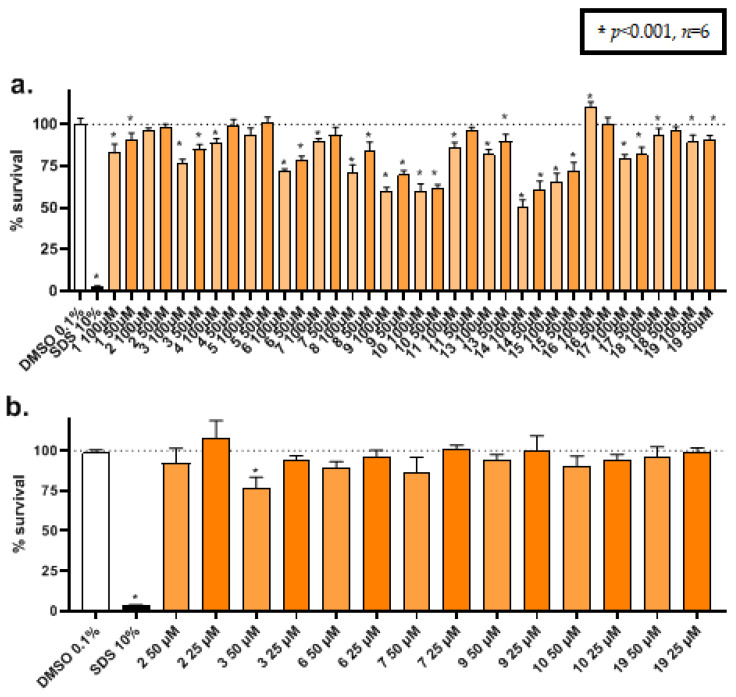
Cytotoxic screening of all compounds in MCF7/S0.5 cells (**a**) and toxicity of selected active compounds in non-cancerous cell line HK-2 (**b**) at two different lower concentrations. Results are shown as the mean ± SD. of three independent experiments. As vehicle was used DMSO 0.1% and sodium dodecyl sulfate 10% (SDS 10%) as negative control. Concentrations of 100 μM correspond to weight concentrations of 46 to 54 μg/mL, depending on the molecular weight. Concentrations of 50 μM correspond to 23 to 27 μg/mL and 25 μM to 11–14 μg/mL. Statistical analysis was performed with one-way ANOVA in order to compare the results to the vehicle (100% viability).

Cytotoxic effect of all the derivates was assessed in sensitive cancerous cell line MCF7/S0.5 at two high concentrations (100 and 50 µM) (Figure 6a). As expected, the survival at concentration 100 µM is lower than that at concentration 50 µM, but in most compounds viability is >75% at 50 µM. In the next step, HK-2 cells were used to evaluate the safety of active compounds at concentrations similar to their IC_50_ in biological assays. Therefore, concentrations of 50 and 25 µM were chosen. As shown in Figure 6b, all compounds showed to be safe, even at the highest concentration, with the exception of compound **3**. As expected, compounds **9** and **10** 50 µM were less toxic in HK-2 cells than in MCF7/S0.5 cells. On the contrary, compound **3** was slightly more toxic in HK-2 cells than in cancerous cells. These data confirm that these compounds can be considered safe at least in vitro models, supporting the relevance of the data reported in this study.

## 3. Materials and Methods

### 3.1. Biological Valuation

#### 3.1.1. Antimicrobial Activity

Evaluation of antimicrobial activity was performed as described previously [76,77]. *Escherichia coli* (ATCC 35210), *Pseudomonas aeruginosa* (ATCC 27853), *Salmonella Typhimurium* (ATCC 13311), and the Gram-positive bacteria *Listeria monocytogenes* (NCTC 7973), *Bacillus cereus* (clinical isolate), and *Staphylococcus aureus* (ATCC 6538) were used as examples of G- bacteria. For comparison, resistant strains methicillin-resistant *Staphylococcus aureus* (IBRS MRSA 011), resistant *Escherichia coli* (IBRS E003) and resistant *Pseudomonas aeruginosa* (IBRS P001) were also employed. The organisms were obtained from the Mycological Laboratory, Department of Plant Physiology, Institute for Biological Research “Siniša Stankovic”—National Institute of Republic of Serbia, Belgrade, Serbia.

The microdilution method was used for the assessment of minimum inhibitory (MIC) and minimum bactericidal (MBC) concentrations. Tested compounds, dissolved in 5% DMSO, were mixed in a Triptic Soy broth (TSB) medium (100 μL) with bacterial inoculum (1.0 × 10^4^ CFU per well) to achieve the planned concentrations (0.001–1.0 mg/mL). The microplates were incubated for 24 h at 37 °C. An addition of 40 μL of iodonitrotetrazolium chloride (0.2 mg/mL) and incubation at 37 °C for 30 min was used for the determination of MIC. MIC was defined as the lowest concentration, producing a significant inhibition of the growth in comparison with the negative control (5% DMSO). Analogously, MBC was assessed by serial sub-cultivations of 10 μL into microplates containing 100 μL of TSB. The lowest concentration that shows no growth after this sub-culturing was determined as the MBC indicating 99.5% death of the original inoculum. Standard, clinically used drugs, streptomycin and ampicillin, were used as positive controls. All experiments were performed in duplicates and repeated three times.

#### 3.1.2. Inhibition of Biofilm Formation

This method was performed as described previously [78], with some modifications. Briefly, a resistant strain of *P. aeruginosa* resistant was incubated with MIC and subMIC of tested compounds in TSB enriched with 2% glucose at 37 °C for 24 h. After this period, each well was washed two times with sterile PBS (Phosphate buffered saline, pH 7.4) and fixed with methanol for 10 min. Methanol was then removed and the plate was air dried. Staining of the biofilm was achieved with 0.1% crystal violet (Bio-Merieux, France) for 30 min. Wells were washed with water and air dried, after an addition of 100 μL of 96% ethanol (Zorka, Serbia). The absorbance was read at 620 nm on a Multiskan™ FC Microplate Photometer, Thermo Scientific™. The percentage of inhibition of biofilm formation was calculated by the following formula:

[(A_620_ control − A_620_ sample)/A_620_ control] × 100. (1)

### 3.2. Antifungal Activity

Six fungal species, Aspergillus niger (ATCC 6275), Aspergillus fumigatus (ATCC 1022), Aspergillus versicolor (ATCC 11730), Penicillium funiculosum (ATCC 36839), Trichoderma viride (IAM 5061), and Penicillium verrucosum var. cyclopium (food isolate) were employed in the antifungal activity testing. The organisms were again obtained from the Mycological Laboratory, Department of Plant Physiology, Institute for Biological Research “Siniša Stankovic,” All experiments were performed in duplicates and repeated three times.

A modified microdilution technique was carried out [79,80]. Briefly, the fungal spores were washed from the surface of agar plates with sterile 0.85% saline containing 0.1% Tween 80 (*v*/*v*). The spore suspension was adjusted with sterile saline to a concentration of approximately 1.0 × 10^5^ in a final volume of 100 μL per well. MIC determinations were carried out by a serial dilution technique using 96-well microtiter plates. The examined compounds were diluted in 5% of DMSO (0.001–1.0 mg/mL) and added in broth Malt medium (MA) with inoculum and incubated for 72 h at 28 °C. The lowest concentrations without visible growth analyzed at the binocular microscope were defined as MICs. The fungicidal concentrations (MFCs) were determined by serial subcultivations of 2 μL of well content into microtiter plates containing 100 μL of broth per well and further incubated for 72 h at 28 °C. MFC was defined as the lowest concentration with no visible growth, suggesting a 99.5% rate of killing of the original inoculum. The clinically used fungicides bifonazole and ketoconazole were used as positive controls (1–3500 µg/mL).

### 3.3. Statistical Analysis

All the assays were carried out three times and the results are expressed as mean values and standard deviation (SD). The results were analyzed using one-way analysis of variance (ANOVA) followed by Tukey’s HSD Test with α = 0.05. The analysis was carried out using the SPSS v. 18.0 program.

### 3.4. Docking

For the docking studies, AutoDock 4.2^®^ software was applied. The X-ray crystal structures data of the used enzymes were taken from the Protein Data Bank (PDB ID: 1KZN, AQGG, 1DDE, JV4T, 2Q85, 1S16 and 5V5Z respectively) following the procedures described in our previous paper [76].

### 3.5. Chemical Similarity Assessment

The Cortellis Drug Discovery Intelligence (CDDI) database [72] contains information about six hundred thousand pharmaceutical agents with more than two million data on experimental pharmacology. The similarity search tool of CDDI is based on the calculation of the structural fingerprints and estimation of the Tanimoto coefficient (TC) [74]. In order to get the data, it is necessary to input the desirable cutoff value of TC; only analogs with the TC value higher of this cutoff will be presented as output data. In this study, we used the default value TC = 80%, which is suggested as the logical cutoff to select the bioactive molecules based on structural similarity [75]. The similarity search was carried out using the MOL file with the structural formula of each from the 19 molecules as a query. As output, we obtained the list of structural formulae of analogs with the additional data on the therapeutic group, mechanism of action, etc.

### 3.6. In-Silico Predictive Studies

Drug-likeness is one important tool employed for predicting drug-like properties. It is designated as an intricate balance of diverse molecular and structural features which plays a pivotal role in establishing whether the specific drug candidate is an oral drug or not. The targeted molecules were appraised for predicting the drug-likeness based on five separate filters namely Egan [81], Ghose [82], Muegge [83], Veber [84] and Lipinski [85] rules accompanying bioavailability and drug-likeness scores using the Molsoft software and SwissADME program (http://swissadme.ch, accessed on 10 May 2021) and using the ChemAxon’s Marvin JS structure drawing tool.

### 3.7. Cytotoxicity Assays

Cellular viability was assessed employing CellTiter 96^®^ Aqueous Non-radioactive Cell Proliferation Assay (Promega, Madison, WI, USA). This method uses the reduction of (3-(4,5-dimethylthiazol-2-yl)-5-(3-carboxymethoxyphenyl)-2-(-(4-sulfophenyl 2*H*-tetrazolium) by viable cells to assess the toxicity of a tested compound. The amount of the derivate formed is measured after an incubation period at a wavelength of 490 nm. Experiments were performed including a vehicle control (DMSO 0.1%), a toxic control (SDS 10%), and the tested compounds at the desired concentrations for 48 h. After the incubation period, 20 μL/well of MTS reagent was added and incubated for a further 3 h. Absorbance was measured using a Hidex Sense Beta Plus plate reader (Hidex, Turku, Finland). Results are expressed as the relative cell viability compared to the vehicle, which was set to 100% viability. All experiments were performed in triplicates and repeated at least three times. MCF7/S0.5 breast cancer cell lines, adapted to a low-sera environment were cultivated in DMEM/F-12 phenol red-free media supplemented with 1% FBS charcoal-stripped and 6 ng/mL insulin, according to the manufacturer guidelines. HK-2, a non-cancerous kidney cell line, was cultivated in DMEM high glucose supplemented with 2 mM L-glutamine.

## 4. Conclusions

Nineteen triazolo-thiadiazole derivatives were evaluated for their activity in inhibiting numerous Gram-positive and Gram-negative bacteria and fungi. The antibacterial activity of compounds (MIC at 0.002–0.150 mg/mL and MBC 0.005–0.200 mg/mL) was higher than those of ampicillin and streptomycin against the tested strains. *E. coli* was found to be the most sensitive strain, whereas the most resistant one was *P. aeruginosa.* Tested compounds also exhibited a good activity against resistant strains, while compound **3** exhibited higher ability to inhibit biofilm formation than both reference drugs.

The activity of tested derivatives against fungi was superior to the reference drugs ketoconazole and bifonazole. The different response of the growth of both Gram-negative and Gram-positive bacteria and fungi towards tested the compounds is probably an indication of either different modes of action due to various substituents or the fact that the metabolism inside bacteria/fungi could have overcome the effect of the compounds or adjusted to it.

Additionally, in relation to pathogenic fungi, the tested compounds possessed very good therapeutic potential against all the fungal species tested, being more active than the clinically used antifungal drugs ketoconazole and bifonazole.

*T. viride* and *A. versicolor* were the most sensitive fungi, while the *A. fumigatus* appeared to be the most resistant one. It should be emphasized that the activity of tested compounds was not equal toward the growth of both Gram-negative and Gram-positive bacteria and fungi. This suggests that different substituents may lead to different modes of action or that the metabolism inside bacteria/fungi could have either overcome the effect of the compounds or adapted to it.

Docking analysis of different bacteria and fungi targets suggested a probable involvement of MurB inhibition in the antibacterial mechanism of most compounds and a probable involvement of MurA inhibition at the mechanism of action of compounds **12**, **13**, and **17**. On the contrary, 14α-lanosterol demethylase (CYP51) is predicted to be a possible mechanism of the antifungal activity of these compounds.

As a result of our study, we have identified the most promising antimicrobial compounds among the nineteen earlier synthesized substances combining triazolo based-thiadiazole and sulfonamide moiety. Antibacterial and antifungal action of the most active compounds **2**, **3**, **6**, **7**, and **19** supersedes those of the reference drugs. Moreover, a similarity search in the CDDI database demonstrates that antimicrobial action is rather new for the investigated chemical series. Therefore, biological activity of the identified potent antimicrobial agents could be recommended for more detailed investigations both in in vitro and in vivo assays.

## Figures and Tables

**Table 1:** Structure of tested compounds.

Compounds Number	Structure	Compounds	Structure
**1**	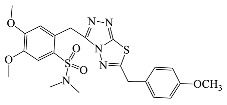	**10**	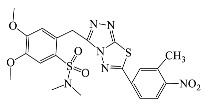
**2**	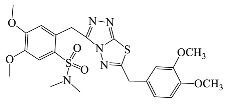	**11**	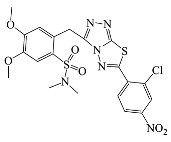
**3**	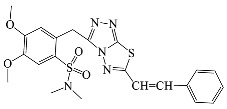	**12**	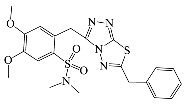
**4**	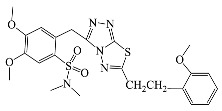	**13**	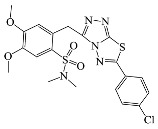
**5**	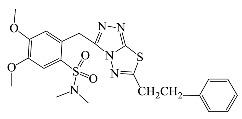	**14**	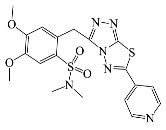
**6**	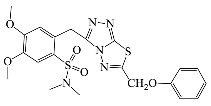	**15**	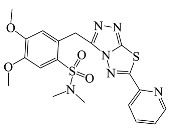
**7**	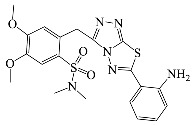	**16**	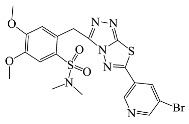
**8**	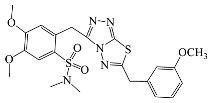	**17**	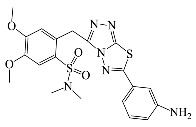
**9**	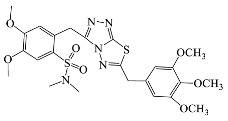	**18**	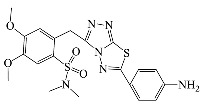
		**19**	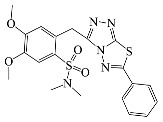

**Table 2:** Antibacterial activity of compounds **1**–**19** (MIC and MBC in μg/mL).

Compounds		*B.c*	*S.a*	*L.m.*	*P.a.*	*E. coli*	*S.t*
**1**	**MIC**	20 ± 0.000	15 ± 0.004	15 ± 0.004	150 ± 0.04	5 ± 0.000	15 ± 0.002
	**MBC**	40 ± 0.000	40 ± 0.000	20 ± 0.008	200 ± 0.070	10 ± 0.00	20 ± 0.000
**2**	**MIC**	15 ± 0.002	10 ± 0.000	15 ± 0.004	20 ± 0.000	5 ± 0.000	15 ± 0.004
	**MBC**	20 ± 0.000	20 ± 0.000	20 ± 0.000	40 ± 0.000	10 ± 0.000	20 ± 0.004
**3**	**MIC**	15 ± 0.004	30 ± 0.007	5 ± 0.000	15 ± 0.000	5 ± 0.000	10 ± 0.000
	**MBC**	20 ± 0.004	40 ± 0.006	10 ± 0.000	20 ± 0.000	10 ± 0.000	20 ± 0.000
**4**	**MIC**	15 ± 0.002	10 ± 0.000	15 ± 0.000	30 ± 0.008	10 ± 0.000	10 ± 0.000
	**MBC**	20 ± 0.004	0.20 ± 0.002	0.020 ± 0.002	0.040 ± 0.000	0.020 ± 0.000	0.020 ± 0.000
**5**	**MIC**	15 ± 0.002	20 ± 0.000	30 ± 0.000	15 ± 0.004	5 ± 0.000	3 ± 0.005
	**MBC**	20 ± 0.002	36 ± 0.004	40 ± 0.000	20 ± 0.000	10 ± 0.000	40 ± 0.000
**6**	**MIC**	10 ± 0.000	10 ± 0.000	15 ± 0.002	20 ± 0.000	8 ± 0.000	5 ± 0.000
	**MBC**	20 ± 0.000	20 ± 0.000	36 ± 0.004	40 ± 0.000	10 ± 0.000	10 ± 0.000
**7**	**MIC**	15 ± 0.002	20 ± 0.000	15 ± 0.002	15 ± 0.004	5 ± 0.000	10 ± 0.000
	**MBC**	20 ± 0.000	40 ± 0.000	20 ± 0.000	20 ± 0.000	20 ± 0.000	20 ± 0.000
**8**	**MIC**	20 ± 0.000	20 ± 0.000	20 ± 0.000	40 ± 0.000	10 ± 0.000	10 ± 0.000
	**MBC**	36 ± 0.005	40 ± 0.000	40 ± 0.000	73 ± 0.009	20 ± 0.000	20 ± 0.000
**9**	**MIC**	20 ± 0.000	30 ± 0.000	10 ± 0.000	23 ± 0.004	5 ± 0.000	10 ± 0.000
	**MBC**	40 ± 0.000	40 ± 0.000	20 ± 0.000	40 ± 0.000	10 ± 0.000	20 ± 0.000
**10**	**MIC**	15 ± 0.002	10 ± 0.000	23 ± 0.004	40 ± 0.000	8 ± 0.000	8 ± 0.000
	**MBC**	20 ± 0.000	20 ± 0.000	40 ± 0.000	60 ± 0.000	10 ± 0.000	10 ± 0.000
**11**	**MIC**	10 ± 0.000	20 ± 0.000	10 ± 0.000	40 ± 0.000	5 ± 0.000	10 ± 0.000
	**MBC**	23 ± 0.004	40 ± 0.000	20 ± 0.000	67 ± 0.009	10 ± 0.000	20 ± 0.000
**12**	**MIC**	20 ± 0.000	20 ± 0.000	10 ± 0.000	80 ± 0.000	5 ± 0.000	8 ± 0.000
	**MBC**	40 ± 0.000	37 ± 0.005	20 ± 0.000	150 ± 0.000	10 ± 0.000	10 ± 0.000
**13**	**MIC**	10 ± 0.000	20 ± 0.000	10 ± 0.000	150 ± 0.020	3 ± 0.000	10 ± 0.000
	**MBC**	20 ± 0.000	40 ± 0.000	20 ± 0.000	200 ± 0.000	47 ± 0.009	20 ± 0.000
**14**	**MIC**	15 ± 0.002	40 ± 0.000	40 ± 0.000	40 ± 0.000	8 ± 0.000	40 ± 0.000
	**MBC**	20 ± 0.000	60 ± 0.000	80 ± 0.000	80 ± 0.000	13 ± 0.002	20 ± 0.000
**15**	**MIC**	5 ± 0.000	20 ± 0.000	15 ± 0.002	36 ± 0.004	8 ± 0.000	15 ± 0.000
	**MBC**	10 ± 0.000	40 ± 0.000	20 ± 0.000	60 ± 0.000	10 ± 0.000	20 ± 0.000
**16**	**MIC**	15 ± 0.000	40 ± 0.000	10 ± 0.000	67 ± 0.009	5 ± 0.000	10 ± 0.000
	**MBC**	20 ± 0.000	80 ± 0.000	20 ± 0.000	80 ± 0.000	10 ± 0.000	20 ± 0.000
**17**	**MIC**	8 ± 0.000	15 ± 0.004	10 ± 0.000	40 ± 0.000	5 ± 0.000	10 ± 0.000
	**MBC**	20 ± 0.000	40 ± 0.000	20 ± 0.000	80 ± 0.000	10 ± 0.000	20 ± 0.007
**18**	**MIC**	15 ± 0.002	30 ± 0.007	10 ± 0.000	37 ± 0.005	5 ± 0.000	10 ± 0.000
	**MBC**	20 ± 0.000	40 ± 0.000	20 ± 0.000	60 ± 0.000	10 ± 0.000	20 ± 0.000
**19**	**MIC**	5 ± 0.000	10 ± 0.000	10 ± 0.000	20 ± 0.000	5 ± 0.000	5 ± 0.000
	**MBC**	10 ± 0.000	20 ± 0.000	20 ± 0.000	40 ± 0.000	10 ± 0.000	10 ± 0.000
**Streptomyci**	**MIC**	25 ± 0.000	100 ± 0.000	150 ± 0.000	100 ± 0.000	100 ± 0.000	100 ± 0.000
	**MBC**	50 ± 0.000	200 ± 0.010	300 ± 0.010	200 ± 0.010	200 ± 0.000	200 ± 0.010
**Ampicillin**	**MIC**	100 ± 0.000	100 ± 0.000	150 ± 0.000	300 ± 0.010	150 ± 0.000	100 ± 0.000
	**MBC**	150 ± 0.000	150 ± 0.000	300 ± 0.020	500 ± 0.010	200 ± 0.010	200 ± 0.000

MIC, minimal inhibitory concentration; MBC, minimal bactericidal concentration; *B.c, Bacilus cereus*; *S.a*., *S. aurues* (ATCC 6538); *l.m*., *L. monocytogenes* (NCTC 7973); *P.a*., *P. aeruginosa* (ATCC 27853); *E. coli*, *E. coli* (ATTC 35210); *S. t*, *S. typhimirium* (ATCC 13311).

**Table 3:** Antibacterial activity of compounds **1**–**19** against resistant strains (MIC and MBC in μg/mL).

Compounds		*MRSA*	*P.a*.	*E. coli*	Compounds		*MRSA*	*P.a.*	*E. coli*
**1**	**MIC**	20 ± 0.000	10 ± 0.000	10 ± 0.000	**11**	**MIC**	10 ± 0.000	8 ± 0.000	10 ± 0.000
	**MBC**	40 ± 0.000	20 ± 0.000	20 ± 0.000		**MBC**	20 ± 0.007	10 ± 0.000	20 ± 0.000
**2**	**MIC**	30 ± 0.007	10 ± 0.000	10 ± 0.000	**12**	**MIC**	20 ± 0.000	10 ± 0.000	20 ± 0.000
	**MBC**	40 ± 0.000	20 ± 0.000	20 ± 0.000		**MBC**	40 ± 0.000	20 ± 0.000	37 ± 0.005
**3**	**MIC**	30 ± 0.007	15 ± 0.002	15 ± 0.002	**13**	**MIC**	30 ± 0.007	10 ± 0.000	20 ± 0.000
	**MBC**	40 ± 0.000	40 ± 0.000	40 ± 0.000		**MBC**	40 ± 0.000	20 ± 0.000	40 ± 0.000
**4**	**MIC**	30 ± 0.007	10 ± 0.000	10 ± 0.000	**14**	**MIC**	67 ± 0.009	40 ± 0.000	30 ± 0.007
	**MBC**	40 ± 0.000	20 ± 0.000	20 ± 0.000		**MBC**	80 ± 0.000	80 ± 0.000	40 ± 0.000
**5**	**MIC**	20 ± 0.004	20 ± 0.004	20 ± 0.000	**15**	**MIC**	15 ± 0.002	80 ± 0.000	10 ± 0.000
	**MBC**	40 ± 0.000	40 ± 0.000	40 ± 0.000		**MBC**	20 ± 0.000	150 ± 0.002	20 ± 0.000
**6**	**MIC**	20 ± 0.0002	15 ± 0.002	15 ± 0.002	**1** **6**	**MIC**	20 ± 0.000	2 ± 0.000	15 ± 0.002
	**MBC**	40 ± 0.000	20 ± 0.000	20 ± 0.000		**MBC**	40 ± 0.000	5 ± 0.000	20 ± 0.000
**7**	**MIC**	20 ± 0.000	10 ± 0.000	10 ± 0.000	**17**	**MIC**	15 ± 0.002	5 ± 0.000	10 ± 0.000
	**MBC**	40 ± 0.000	20 ± 0.001	20 ± 0.000		**MBC**	20 ± 0.000	10 ± 0.000	20 ± 0.000
**8**	**MIC**	80 ± 0.002	10 ± 0.000	10 ± 0.000	**18**	**MIC**	20 ± 0.000	40 ± 0.000	10 ± 0.000
	**MBC**	150 ± 0.010	20 ± 0.000	20 ± 0.008		**MBC**	40 ± 0.000	73 ± 0.009	20 ± 0.000
**9**	**MIC**	30 ± 0.000	10 ± 0.000	10 ± 0.000	**19**	**MIC**	20 ± 0.000	5 ± 0.000	15 ± 0.000
	**MBC**	40 ± 0.000	20 ± 0.008	20 ± 0.000		**MBC**	40 ± 0.000	10 ± 0.000	20 ± 0.000
**10**	**MIC**	10 ± 0.001	15 ± 0.005	15 ± 0.004	**Streptomycin**	**MIC**	100 ± 0.000	50 ± 0.000	100 ± 0.000
	**MBC**	20 ± 0.000	20 ± 0.008	20 ± 0.000		**MBC**	-	100 ± 0.000	200 ± 0.010
					**Ampicillin**	**MIC**	-	200 ± 0.010	200 ± 0.010
						**MBC**	-	-	-

MIC, minimal inhibitory concentration; MBC, minimal bactericidal concentration; MRSA, methicillin resistant *S. aureus*, (IBRS MRSA 011); *E. coli* res, resistant *E. coli* (IBRS E003); *P.a.* res, resistant *P. aeruginosa* (IBRS P001).

**Table 4:** Effect of selected compounds on *P. aeruginosa* biofilm formation.

Compound	MIC	0.5 MIC
Biofilm inhibition (% compared to no treatment)		
**2**	49.82 ± 2.35	40.74 ± 8.89
**3**	75.10 ± 6.89	62.82 ± 4.56
**6**	50.85 ± 8.82	31.90 ± 6.98
**7**	44.13 ± 3.56	38.75 ± 2.11
**19**	47.84 ± 2.36	41.50 ± 1.08
**Ampicillin**	70.00 ± 10.23	52.36 ± 3.67
**Streptomycin**	63.56 ± 8.28	29.12 ± 1.22

**Table 5:** Antifungal activity of compounds **1**–**19**. (ΜIC and MFC in μg/mL).

Compounds		*A.f*	*A.v.*	*A.n.*	*T.v.*	*P.f.*	*P.v.c.*
**1**	**MIC**	5 ± 0.000	2 ± 0.000	10 ± 0.000	2 ± 0.000	20 ± 0.000	10 ± 0.000
	**MFC**	10 ± 0.000	5 ± 0.000	20 ± 0.000	5 ± 0.000	40 ± 0.000	20 ± 0.000
**2**	**MIC**	10 ± 0.000	5 ± 0.000	10 ± 0.000	2 ± 0.000	10 ± 0.000	10 ± 0.000
	**MFC**	20 ± 0.000	10 ± 0.000	20 ± 0.000	5 ± 0.000	20 ± 0.007	20 ± 0.000
**3**	**MIC**	20 ± 0.000	10 ± 0.000	15 ± 0.002	2 ± 0.000	20 ± 0.000	20 ± 0.000
	**MFC**	40 ± 0.000	20 ± 0.000	20 ± 0.002	5 ± 0.000	36 ± 0.004	36 ± 0.004
**4**	**MIC**	2 ± 0.000	2 ± 0.000	5 ± 0.000	5 ± 0.000	10 ± 0.000	10 ± 0.000
	**MFC**	5 ± 0.000	5 ± 0.000	1 ± 0.000	1 ± 0.000	20 ± 0.000	20 ± 0.000
**5**	**MIC**	20 ± 0.000	10 ± 0.000	15 ± 0.002	8 ± 0.000	33 ± 0.005	20 ± 0.000
	**MFC**	36 ± 0.007	20 ± 0.070	20 ± 0.000	10 ± 0.000	40 ± 0.000	40 ± 0.000
**6**	**MIC**	5 ± 0.000	5 ± 0.000	5 ± 0.000	2 ± 0.000	10 ± 0.000	10 ± 0.000
	**MFC**	10 ± 0.000	10 ± 0.000	10 ± 0.000	5 ± 0.000	20 ± 0.000	20 ± 0.000
**7**	**MIC**	10 ± 0.000	10 ± 0.000	5 ± 0.000	5 ± 0.000	5 ± 0.000	20 ± 0.000
	**MFC**	20 ± 0.000	20 ± 0.000	10 ± 0.000	10 ± 0.000	10 ± 0.000	36 ± 0.004
**8**	**MIC**	10 ± 0.000	10 ± 0.000	15 ± 0.002	5 ± 0.000	10 ± 0.000	15 ± 0.002
	**MFC**	20 ± 0.007	20 ± 0.000	20 ± 0.000	10 ± 0.000	20 ± 0.000	20 ± 0.000
**9**	**MIC**	5 ± 0.000	5 ± 0.000	10 ± 0.000	5 ± 0.000	36 ± 0.005	30 ± 0.007
	**MFC**	10 ± 0.000	10 ± 0.000	20 ± 0.002	10 ± 0.000	80 ± 0.000	40 ± 0.000
**10**	**MIC**	32 ± 0.004	20 ± 0.000	10 ± 0.000	5 ± 0.000	10 ± 0.000	20 ± 0.000
	**MFC**	40 ± 0.000	40 ± 0.000	20 ± 0.000	10 ± 0.000	20 ± 0.000	40 ± 0.000
**11**	**MIC**	20 ± 0.000	5 ± 0.000	20 ± 0.000	2 ± 0.001	2 ± 0.000	2 ± 0.000
	**MFC**	40 ± 0.000	10 ± 0.000	32 ± 0.005	5 ± 0.000	5 ± 0.000	5 ± 0.000
**12**	**MIC**	5 ± 0.000	10 ± 0.000	15 ± 0.002	5 ± 0.000	40 ± 0.000	20 ± 0.000
	**MFC**	20 ± 0.000	20 ± 0.000	20 ± 0.000	10 ± 0.003	67 ± 0.009	40 ± 0.000
**13**	**MIC**	40 ± 0.000	20 ± 0.007	20 ± 0.000	2 ± 0.000	40 ± 0.000	32 ± 0.005
	**MFC**	67 ± 0.009	36 ± 0.005	40 ± 0.000	5 ± 0.000	80 ± 0.000	40 ± 0.000
**14**	**MIC**	10 ± 0.000	10 ± 0.000	10 ± 0.000	5 ± 0.000	30 ± 0.007	32 ± 0.005
	**MFC**	36 ± 0.007	20 ± 0.000	20 ± 0.001	10 ± 0.003	40 ± 0.000	40 ± 0.000
**15**	**MIC**	15 ± 0.000	10 ± 0.000	20 ± 0.000	5 ± 0.000	30 ± 0.007	30 ± 0.007
	**MFC**	20 ± 0.000	20 ± 0.000	37 ± 0.005	10 ± 0.000	40 ± 0.000	36 ± 0.005
**16**	**MIC**	20 ± 0.000	10 ± 0.000	5 ± 0.000	5 ± 0.000	10 ± 0.000	20 ± 0.000
	**MFC**	40 ± 0.000	20 ± 0.000	1 ± 0.000	8 ± 0.000	20 ± 0.000	40 ± 0.000
**17**	**MIC**	20 ± 0.000	20 ± 0.000	20 ± 0.000	5 ± 0.000	30 ± 0.007	30 ± 0.007
	**MFC**	40 ± 0.000	40 ± 0.000	32 ± 0.005	10 ± 0.003	40 ± 0.000	40 ± 0.000
**18**	**MIC**	20 ± 0.000	10 ± 0.000	10 ± 0.000	8 ± 0.000	20 ± 0.000	30 ± 0.007
	**MFC**	32 ± 0.004	20 ± 0.000	20 ± 0.000	10 ± 0.000	40 ± 0.000	36 ± 0.005
**19**	**MIC**	20 ± 0.000	20 ± 0.000	10 ± 0.000	8 ± 0.000	20 ± 0.000	20 ± 0.000
	**MFC**	40 ± 0.000	36 ± 0.005	20 ± 0.000	10 ± 0.000	40 ± 0.000	40 ± 0.000
**Ketoconazole**	**MIC**	20 ± 0.010	200 ± 0.000	200 ± 0.010	1000 ± 0.010	200 ± 0.000	200 ± 0.010
	**MFC**	500 ± 0.030	500 ± 0.020	500 ± 0.030	1500 ± 0.020	500 ± 0.030	300 ± 0.010
**Bifonazole**	**MIC**	150 ± 0.000	100 ± 0.000	150 ± 0.000	150 ± 0.000	200 ± 0.010	100 ± 0.000
	**MFC**	200 ± 0.000	200 ± 0.010	200 ± 0.000	200 ± 0.010	250 ± 0.010	200 ± 0.000

*A.v*., *A. versicolor* (ATCC 11730); *T.v*., *T. viride* (IAM 5061); *A.n*., *A. niger* (ATCC 6275); *P.v.c*., *Penicillium verrucosum* var. *cyclopium* (food isolate); *P.f*., *P. funiculosum* (ATCC 36839); *A.f*., *A. fumigatus* (human isolate).

**Table 6:** Molecular docking results to antibacterial targets.

Comp.	Est. Binding Energy (kcal/mol)					I-H * E. coli MurB	Residues *E. coli* MurB
	*E. coli* Gyrase 1KZN	Thymidylate kinase 4QGG	*E. coli* Primase 1DDE	*E. coli* MurA JV4T	*E. coli* MurB 2Q85		
**1**	−4.45	−3.12	-	-	−7.15	2	Arg158, Ser228
**2**	−2.89	-	-	−6.19	−10.92	3	Arg158, Ser228, Asn232
**3**	−5.32	−1.51	−6.29	−5.27	−12.18	3	Arg158, Arg213, Ser228
**4**	−6.95	−1.22	−4.28	−5.33	−8.03	2	Arg158, Ser228
**5**	−2.39	−1.18	−2.44	−6.01	−9.21	2	Arg213, Ser228
**6**	−4.78	-	−4.93	−5.88	−12.10	3	Gly122, Arg213, Ser228
**7**	−3.16	-	−5.36	−5.32	−10.23	3	Arg213, Ser228, Lys261
**8**	-	−3.22	-	−6.28	−8.62	2	Arg213, Ser228
**9**	−4.77	−2.13	−3.95	-	−10.07	3	Arg213, Ser228, Asn232
**10**	−4.01	-	−2.55	−2.53	−10.11	3	Arg213, Ser228
**11**	−5.51	−3.25	-	-	−9.92	3	Arg158, Arg213, Ser228
**12**	−5.14	−2.99	-	-	−10.03	3	Arg213, Ser228
**13**	−3.12	-	-	−4.83	−7.75	2	Arg213, Ser228
**14**	−2.17	-		-	−7.77	2	Arg158, Ser228
**15**	-	-	−2.09	−2.25	−8.94	2	Arg213, Ser228
**16**	-	-	−3.85	−6.94	−8.13	2	Ser228, Lys261
**17**	−4.28	−4.11	-	−5.71	−8.74	2	Arg158, Ser228
**18**	-	−3.15	−4.16	−3.82	−9.71	2	Arg213, Ser228
**19**	−5.13	−5.02	−6.11	−6.33	−13.56	4	Arg213, Ser228, Lys261

* I-H: Number of hydrogen bonds.

**Table 7:** Molecular docking results on antifungal targets.

	Est. Binding Energy(kcal/mol)				
N/N	DNA TopoIV 1S16	CYP51 of *C. albicans* *5V5Z*	I-H	Residues CYP51 of *C. albicans*	Interactions with HEM601
**1**	−4.16	−7.96	1	Tyr132	Hydrophobic
**2**	−2.15	−7.88	1	Tyr64	-
**3**	−3.19	−7.63	1	Tyr118	-
**4**	−2.88	−8.63	-	-	aromatic
**5**	-	−7.55	1	Tyr118	Hydrophobic
**6**	−5.29	−8.11	1	Tyr64	-
**7**	−3.36	−8.07	1	Tyr132	-
**8**	-	−8.15	1	Tyr118	Hydrophobic
**9**	-	−7.07	1	Tyr132	-
**10**	−2.58	−7.13	1	Tyr132	Hydrophobic
**11**	−3.95	−7.03	1	Tyr118	-
**12**	-	−6.87	1	Tyr118	-
**13**	−3.77	−6.96	1	Tyr132	-
**14**	−4.55	−8.26	1	Tyr132	Hydrophobic
**15**	-	−8.22	1	Met508	Hydrophobic
**16**	−1.12	−7.31	1	Tyr132	-
**17**	−3.74	−7.59	1	Tyr118	-
**18**	−5.19	−8.22	1	Tyr132	Hydrophobic
**19**	−1.52	−7.91	-	-	Hydrophobic, aromatic
**ketoconazole**	-	−8.23	1	Tyr64	Hydrophobic, aromatic

**Table 8:** Drug likeness predictions and Physicochemical-Pharmacokinetic/ADME properties of tested compounds.

No	MW	Number of HBA ^a^	Number of HBD ^b^	Log *P*_o/w_ (iLOGP) ^c^	Log S ^d^	TPSA ^e^	BBB Permeant ^f^	Lipinski, Ghose, Veber, Egan, and Muegge Violations	Bioavailability Score	Drug-Likeness Model Score
**1**	503.59	9	0	3.31	Moderately soluble	144.77	No	1	0.55	0.56
**2**	533.62	10	0	3.23	Poorly soluble	154.00	No	2	0.17	0.26
**3**	485.58	8	0	3.09	Poorly soluble	135.54	No	0	0.55	0.53
**4**	517.62	9	0	3.37	Poorly soluble	144.77	No	1	0.55	0.45
**5**	487.60	8	0	3.36	Poorly soluble	135.54	No	0	0.55	0.71
**6**	489.57	9	0	3.54	Poorly soluble	144.77	No	0	0.55	0.49
**7**	474.56	8	1	2.98	Moderately soluble	161.56	No	0	0.55	0.32
**8**	503.59	9	0	2.55	Poorly soluble	144.77	No	1	0.55	0.42
**9**	563.65	11	0	3.96	Poorly soluble	163.22	No	2	0.17	0.14
**10**	518.57	10	0	3.30	Moderately soluble	181.36	No	2	0.17	−0.34
**11**	538.98	10	0	2.91	Poorly soluble	181.36	No	2	0.17	−0.10
**12**	473.57	8	0	3.18	Poorly soluble	135.54	No	0	0.55	0.56
**13**	493.99	8	0	3.48	Poorly soluble	135.54	No	0	0.55	0.94
**14**	460.53	9	0	2.72	Moderately soluble	148.46	No	0	0.55	0.89
**15**	460.53	9	0	2.92	Moderately soluble	148.43	No	0	0.55	0.67
**16**	539.43	9	0	2.80	Poorly soluble	148.43	No	1	0.55	0.21
**17**	474.56	8	1	2.85	Moderately soluble	161.56	No	0	0.55	0.43
**18**	474.56	8	1	2.78	Moderately soluble	161.56	No	0	0.55	0.76
**19**	459.54	8	0	3.14	Poorly soluble	135.54	No	0	0.55	0.51

^a^ number of hydrogen bond acceptors; ^b^ number of hydrogen bond donors; ^c^ lipophilicity; ^d^ Water solubility (SILICOS-IT [S = Soluble]); ^e^ topological polar surface area (Å^2^); ^f^ Blood Brain Barrier permeant.

## Data Availability

Not applicable.

## Supplementary Materials

The following are available online at https://www.mdpi.com/article/10.3390/antibiotics10070804/s1, Table S1. Prediction of toxicity by ToxPredict program; Table S2. Prediction of toxicity by PROTOX program.
