# Supplementary material for: Triazolo Based-Thiadiazole Derivatives. Synthesis, Biological Evaluation and Molecular Docking Studies
Source: Antibiotics (Basel). 2021 Jul 2;10(7):804. doi: 10.3390/antibiotics10070804 (PMC8300616; doi:10.3390/antibiotics10070804)
Supplement: Supplementary file 1 [file antibiotics-10-00804-s001.zip › antibiotics-1241051-supplementary.pdf]

antibiotics

MDPI

Article

Triazolo Based-Thiadiazole Derivatives. Synthesis, Biological Evaluation and Molecular Docking Studies

Charalampos Kamoutsis [1], Maria Fesatidou [2], Anthi Petrou [2], Athina Geronikaki [2,*], Vladimir Poroikov [3], Marija Ivanov [4], Marina Soković [4], Ana Ćirić [4], Alejandro Carazo [5] and Přemysl Mladěnka [5]

[1] School of Pharmacy, University of Patras, 26504 Patras, Greece; kamoutsi@upatras.gr
[2] School of Pharmacy, Aristotle University of Thessaloniki, 54124 Thessaloniki, Greece; marifesa@pharm.auth.gr (M.F.); aipetrou@pharm.auth.gr (A.P.)
[3] Institute of Biomedical Chemistry, Laboratory of Structure-Function Drug Design, Pogodinskaya str. 10, Bldg. 8, 119121 Moscow, Russia; vladimir.poroikov@ibmc.msk.ru
[4] Institute for Biological Research "Siniša Stanković"—National Institute of Republic of Serbia, University of Belgrade, Blvd. despot Stefan 142, 11000 Belgrade, Serbia; marija.smiljkovic@ibiss.bg.ac.rs (M.I.); mris@ibiss.bg.ac.rs (M.S.); rancic@ibiss.bg.ac.rs (A.Ć.)
[5] Department of Pharmacology and Toxicology, Faculty of Pharmacy, Charles University, Akademika Heyrovského 1203, 500 05 Hradec Králové, Czech Republic; carazofa@faf.cuni.cz (A.C.); mladenkap@faf.cuni.cz (P.M.)
* Correspondence: geronik@pharm.auth.gr; Tel.: +30-23-1099-7616

Table S1. Prediction of toxicity by ToxPredict program.

Com.	Carcinogenicity (Rodents (multiple species/sites))	Carcinogenicity (Rat)	Carcinogenicity (Mouse)	Mutagenicity (Salmonella typhimurium)
1	Inactive Conf.: 0.026	Inactive Conf.: 0.029	Inactive Conf.: 0.028	Inactive Conf.: 0.027
2	Inactive Conf.: 0.027	Inactive Conf.: 0.027	Inactive Conf.: 0.042	Inactive Conf.: 0.043
3	Inactive Conf.: 0.031	Inactive Conf.: 0.042	Inactive Conf.: 0.052	Inactive Conf.: 0.042
4	Inactive Conf.: 0.029	Inactive Conf.: 0.031	Inactive Conf.: 0.031	Inactive Conf.: 0.045
5	Inactive Conf.: 0.029	Inactive Conf.: 0.028	Inactive Conf.: 0.044	Inactive Conf.: 0.029
6	Inactive Conf.: 0.033	Inactive Conf.: 0.023	Inactive Conf.: 0.042	Inactive Conf.: 0.026
7	Inactive Conf.: 0.041	Inactive Conf.: 0.027	Inactive Conf.: 0.045	Inactive Conf.: 0.027
8	Inactive Conf.: 0.029	Inactive Conf.: 0.025	Inactive Conf.: 0.029	Inactive Conf.: 0.027
9	Inactive Conf.: 0.029	Inactive Conf.: 0.026	Inactive Conf.: 0.029	Inactive Conf.: 0.027
10	Inactive Conf.: 0.028	Inactive Conf.: 0.030	Inactive Conf.: 0.031	Inactive Conf.: 0.028
11	Inactive Conf.: 0.031	Inactive Conf.: 0.029	Inactive Conf.: 0.045	Inactive Conf.: 0.029
12	Inactive Conf.: 0.041	Inactive Conf.: 0.028	Inactive Conf.: 0.042	Inactive Conf.: 0.029
13	Inactive Conf.: 0.028	Inactive Conf.: 0.027	Inactive Conf.: 0.028	Inactive Conf.: 0.028
14	Inactive Conf.: 0.031	Inactive Conf.: 0.028	Inactive Conf.: 0.042	Inactive Conf.: 0.029
15	Inactive Conf.: 0.032	Inactive Conf.: 0.028	Inactive Conf.: 0.044	Inactive Conf.: 0.029
16	Inactive Conf.: 0.041	Inactive Conf.: 0.029	Inactive Conf.: 0.042	Inactive Conf.: 0.028
17	Inactive Conf.: 0.027	Inactive Conf.: 0.028	Inactive Conf.: 0.029	Inactive Conf.: 0.027
18	Inactive Conf.: 0.027	Inactive Conf.: 0.029	Inactive Conf.: 0.029	Inactive Conf.: 0.029
19	Inactive Conf.: 0.027	Inactive Conf.: 0.029	Inactive Conf.: 0.032	Inactive Conf.: 0.032

Table S2. Prediction of toxicity by PROTOX program.

Com.	Predicted LD50 mg/kg	Predict. Toxicity Class	Hepato-toxicity	Carcinogenicity	Mutagenicity	Cytotoxicity	Immuno-toxicity
**1**	800	4	Inactive 0.52	Inactive 0.54	Inactive 0.62	Inactive 0.77	Inactive 0.92
**2**	800	4	Inactive 0.53	Inactive 0.53	Inactive 0.65	Inactive 0.78	Inactive 0.91
**3**	800	4	Inactive 0.51	Inactive 0.53	Inactive 0.62	Inactive 0.81	Inactive 0.98
**4**	800	4	Inactive 0.50	Inactive 0.53	Inactive 0.61	Inactive 0.77	Inactive 0.92
**5**	800	4	Inactive 0.53	Inactive 0.62	Inactive 0.61	Inactive 0.81	Inactive 0.99
**6**	800	4	Inactive 0.50	Inactive 0.51	Inactive 0.64	Inactive 0.82	Inactive 0.96
**7**	800	4	Inactive 0.50	Inactive 0.52	Inactive 0.65	Inactive 0.79	Inactive 0.92

**8**	800	4	Inactive 0.50	Inactive 0.66	Inactive 0.61	Inactive 0.80	Inactive 0.96
**9**	800	4	Inactive 0.52	Inactive 0.50	Inactive 0.60	Inactive 0.78	Inactive 0.97
**10**	800	4	Inactive 0.61	Inactive 0.54	Inactive 0.61	Inactive 0.75	Inactive 0.91
**11**	800	4	Inactive 0.61	Inactive 0.50	Inactive 0.54	Inactive 0.74	Inactive 0.92
**12**	800	4	Inactive 0.54	Inactive 0.60	Inactive 0.64	Inactive 0.70	Inactive 0.90
**13**	800	4	Inactive 0.57	Inactive 0.62	Inactive 0.64	Inactive 0.78	Inactive 0.95
**14**	800	4	Inactive 0.50	Inactive 0.53	Inactive 0.64	Inactive 0.77	Inactive 0.92
**15**	800	4	Inactive 0.51	Inactive 0.54	Inactive 0.62	Inactive 0.78	Inactive 0.92
**16**	800	4	Inactive 0.54	Inactive 0.61	Inactive 0.65	Inactive 0.78	Inactive 0.90
**17**	1400	4	Inactive 0.56	Inactive 0.54	Inactive 0.55	Inactive 0.78	Inactive 0.98
**18**	1400	4	Inactive 0.56	Inactive 0.74	Inactive 0.51	Inactive 0.74	Inactive 0.99
**19**	1400	4	Inactive 0.54	Inactive 0.56	Inactive 0.56	Inactive 0.81	Inactive 0.99
